# Joint estimation over multiple individuals improves behavioural state inference from animal movement data

**DOI:** 10.1038/srep20625

**Published:** 2016-02-08

**Authors:** Ian Jonsen

**Affiliations:** 1 Macquarie University, Department of Biological Sciences, Sydney, NSW, 2109, Australia

## Abstract

State-space models provide a powerful way to scale up inference of movement behaviours from individuals to populations when the inference is made across multiple individuals. Here, I show how a joint estimation approach that assumes individuals share identical movement parameters can lead to improved inference of behavioural states associated with different movement processes. I use simulated movement paths with known behavioural states to compare estimation error between nonhierarchical and joint estimation formulations of an otherwise identical state-space model. Behavioural state estimation error was strongly affected by the degree of similarity between movement patterns characterising the behavioural states, with less error when movements were strongly dissimilar between states. The joint estimation model improved behavioural state estimation relative to the nonhierarchical model for simulated data with heavy-tailed Argos location errors. When applied to Argos telemetry datasets from 10 Weddell seals, the nonhierarchical model estimated highly uncertain behavioural state switching probabilities for most individuals whereas the joint estimation model yielded substantially less uncertainty. The joint estimation model better resolved the behavioural state sequences across all seals. Hierarchical or joint estimation models should be the preferred choice for estimating behavioural states from animal movement data, especially when location data are error-prone.

The study of terrestrial and aquatic animal movements from electronic telemetry data has burgeoned in recent years^1^,^2^. This growth has contributed substantially to the establishment of movement ecology as a rich and fundamental sub-discipline of ecology^3^. One motivation for animal-borne telemetry studies is to understand individual space use patterns in an environmental context, extrapolating these to population-, species- and community-level patterns^4^,^5^,^6^ and predicting responses to possible future environmental change^7^. The location data used in these studies typically is devoid of behavioural context that can inform the relative profitability of habitats encountered, but process-based statistical models can be used to infer some of this context^8^,^9^,^10^.

Behavioural switching models objectively divide movement paths into discrete behavioural states based on an underlying, assumed movement process that is typically, but not necessarily, a correlated random walk^8^,^11^ or a biased correlated random walk^12^. This process-based approach has been implemented as a hidden Markov model^8^,^13^ or a hidden semi-Markov model^14^,^15^ where state variables are discrete, and as a state-space model^16^,^17^ where discrete and continuous state variables can be mixed together. The hidden Markov or hidden semi-Markov models are commonly fit to GPS location data that have negligible measurement error^18^. In contrast, state-space models are more flexible, can be applied to location data that span the gamut of measurement error, and have proven to be particularly useful for Argos satellite^11^,^19^ and light-level geolocation^20^,^21^ datasets.

Bayesian methods have facilitated use of state-space models with complex likelihoods^11^,^22^, but usually with much greater computation time compared to the more tractable, frequentist implementations of hidden Markov and hidden semi-Markov models^14^,^23^. Despite this trade-off, Bayesian methods provide a simpler approach to conduct inference across individuals by permitting a hierarchical structure to the priors on some or all model parameters^24^,^25^. Estimating behavioural states and associated movement parameters across individual animals is potentially important for two reasons. First, it can provide a direct approach for scaling individual movements up to the population-level, for example, to assess among-individual variation in foraging behaviour^22^ and potential environmental correlates^10^. This approach may be the most direct way that movements of individuals can be scaled up to better understand their population dynamics^26^. Second, movement parameters and state variables can be estimated with greater precision by borrowing strength across multiple dataset^24^. Despite these advantages, both Bayesian and frequentist hierarchical implementations of behavioural switching models are not ubiquitous, but see^14^,^25^,^27^,^28^. Only a few evaluations of the efficacy of these behavioural models have been performed^15^,^29^,^30^.

Here I illustrate the benefits of using a state-space model where estimation of behavioural states is conducted jointly across multiple animal movement datasets. The stronger inference conveyed by joint estimation and hierarchical models through aggregation of data is well understood in the quantitative literature^14^,^24^,^31^ but perhaps less so by end users. My aim is to encourage the uptake of such models of animal movement behaviour through a clear exposition of situations in which they may improve behavioural state estimation. I compare the abilities of both a non-hierarchical state-space model (SSM) and a joint estimation state-space model (hSSM) to estimate behavioural states from simulated movement paths where the true states are known. I determine how the behavioural state estimation of both models is affected by: (1) the degree of similarity in movement characteristics between the two behavioural states; and (2) differing levels of location error, mimicking GPS and Argos satellite data. I then compare the abilities of the SSM and hSSM to estimate behavioural states and associated switching parameters from Argos satellite tracking data collected from Weddell seals (*Leptonychotes weddellii*, Lesson).

## Methods

### Movement models

I model movement as a compound CRW that can be decomposed into two or more discrete behavioural states^8^,^11^. For simplicity, the model considers just two states: a transient state consisting of relatively fast and more directionally persistent movements, and an area-restricted search (ARS) state consisting of relatively slow movements with frequent course reversals. The model is a first-difference CRW that includes stochastic switches between behavioural states, where the states are defined as unique combinations of two movement parameters: the mean turn angle 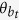 and the move persistence 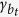. The subscript 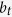 denotes the behavioural state at time *t*, where 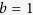 (transient state) or 2 (ARS state). This non-hierarchical model is described elsewhere^11^,^22^,^23^, but has the general form:


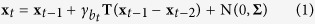


where 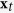 and 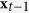 are the unobserved true locations of an animal at times *t* and 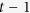. **T** is a matrix describing the mean turn angle, 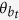, between displacements 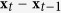 and 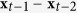:


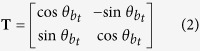


and 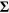 is a variance-covariance matrix specifying the magnitude of stochasticity in the 2-dimensional movements:


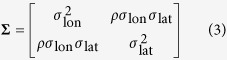


Switching between behavioural states is governed by a Markov chain with fixed transition probabilities:


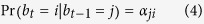


where 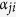 is the probability of switching from behavioural state *j* at time 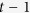 to behavioural state *i* at time *t*. In a 2-state context the *α*_*ji*_’s are elements of a 2 × 2 transition matrix:


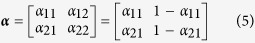


where 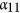 and 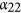 are the probabilities of remaining in the transient and ARS states, respectively. 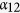 and 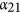 are the probabilities of switching from the transient to the ARS state and from the ARS to the transient state, respectively. These transitions can be estimated assuming a first-order Markov categorical distribution. In practice, only 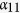 and 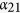 need to be estimated as the rows of ***α*** must sum to 1.

Location uncertainty is accounted for via the observation model. Here I outline two approaches corresponding to the simulation study described below. First, I assume location uncertainty follows a bi-variate Normal distribution, which is typical of GPS data^32^:


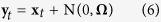


where 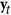 is the observed location at time *t* and **Ω** is a variance-covariance matrix specifying the magnitude of uncertainty in the observed locations.

Second, I assume location uncertainty is heavy-tailed due to occasionally large errors, typical of Argos satellite data, and can be modelled with a generalised *t*-distribution:


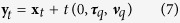


where 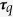 are the scale parameters in the two directions (e.g., longitude and latitude) for 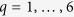 location quality classes, and 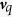 are the associated degrees of freedom. Smaller 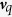 lead to heavier-tailed errors. These parameters can be estimated within the model or separately using independent data with known locations (Table 1)^11^,^33^.

### Hierarchical structure

I fit the model summarised in Eqn's 1–7 as a joint estimation model that assumes individuals share identical movement parameters. This is a simple multi-level structure where the state variables, 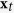 and 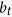, necessarily are estimated at the individual level but movement parameters are estimated among individuals. Hierarchical models more typically assume that (some) movement parameters differ among individuals but are drawn from a set of distributions shared among individuals, thus the parameters would be estimated both within and among individuals^14^,^25^. For convenience, I refer to the simpler joint estimation model considered here as a hierarchical model.

In the Bayesian paradigm, the model can, in principle, easily be implemented in either non-hierarchical or hierarchical form. The non-hierarchical form amounts to fitting the same model separately to each animal track, whereas the hierarchical form assumes that some or all of the model parameters can be shared across individual animals because there are fundamental similarities in their movement behaviours. Typically, a hierarchical model includes 1 or more common distributions from which individual-level parameters are drawn - the random effects. A simpler alternative is to consider a joint estimation model, where key movement parameters are assumed to be identical among individuals. These parameters are not estimated at the individual level, but rather across all individuals. If the analysis is focused on estimating the movement parameters 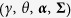 then the joint estimation model may not be realistic or particularly useful as all individuals are painted with the same brush. If, however, the analysis is focused on estimating the behavioural states and their associated locations then the joint estimation model may prove useful.

### Simulations

To evaluate the relative efficacies of hierarchical and nonhierarchical models in estimating behavioural states, I simulated movement paths using the switching CRW model described in Eqn's 1–7. I examined the following scenarios in a factorial manner: large versus small difference in move persistence, *γ*, between behavioural states; and three levels of uncertainty in the location data simulating no measurement error, GPS errors (constant errors) and heavy-tailed, time-varying Argos errors (Table 1). Fifty replicate movement paths each consisting of 200 locations were simulated for each of the six scenarios (see Fig. S1 for examples). I fit a hSSM that builds on the SSM in^11^ and has been used and augmented in previous studies^4^,^10^,^17^,^22^, simultaneously to the 50 movement paths under each scenario. For comparison, I also fit the non-hierarchical SSM to each of these movement paths individually under each scenario. The models are identical except that the key movement parameters 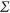, 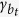, and 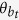 are estimated separately for each individual in the SSM and across all individuals in the hSSM (see Supplementary Information for model code).

I used the root-mean-squared error (RMSE) between the estimated and simulated (truth) behavioural states to assess the relative efficacies of the SSM and hSSM in discriminating the behavioural states. The posterior means, which range continuously between 1 (transient state) and 2 (ARS state), were used to summarise the behavioural state estimates. The Kappa statistic^34^ provides an alternate approach for this assessment, and was used in a similar context by^29^, but this necessitates using the posterior median, which can be 1 or 2 (1.5 is rare), to compare with the simulated behavioural states. Doing so ignores the estimation uncertainty in the behavioural states, which can be summarised via the posterior mean; estimates close to 1 or 2 have low uncertainty whereas estimates close to 1.5 have high uncertainty. Thus the RMSE of the posterior mean behavioural states can capture differences in estimation uncertainty between the two models.

### Comparisons using Weddell seal data

The simulations are convenient for evaluating classification accuracy against truth but they do not capture the full statistical properties of real movement data. Real animal movement data often have time-varying measurement errors, irregularly timed observations, diverse run lengths of underlying behaviour, and behaviours that may not be captured by the movement model being fit. To assess whether a hierarchical formulation might lead to improved inference of unobservable behavioural states, I compared fits of the SSM and hSSM to 10 adult female Weddell seal tracks. Argos location data were collected via Satellite-Relayed Data Loggers (SRDLs), manufactured by the Sea Mammal Research Unit (SMRU, University of St Andrews, Scotland, UK), deployed on the seals. The location data (N = 6 578 Argos locations, spanning approximately 3 months: 11/03/2011 to 27/06/2011) form part of the Australian Integrated Marine Observing System (IMOS) 2011 deployments at Davis station, Antarctica^17^ and are publicly available (http://imos.aodn.org.au).

I restricted the analysis to deployment periods that had data gaps no longer than 4 days. This resulted in two tracks (wd04-881-11, wd04-884-11) being truncated slightly as locations were observed sparsely toward the end of their deployments. On average, the tracks consisted of 659 locations (range: 243, 889), with 8.5 locations day^−1^ (range: 6.6, 10.9).

Due to the irregular sampling of locations that is inevitable with Argos data from diving marine animals the observation model for both the SSM and hSSM differed from that used for the simulated data (Eqn 5). Here the observation model includes a regularisation that links the irregularly timed observations to the states that occur regularly through time^11^:


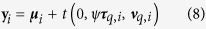


where 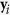 is the 
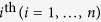
 observed location with the time interval 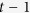 to *t*, 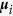 is an estimate of the corresponding true location, 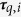 and 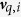 are the scale and df parameters, from a generalised *t*-distribution, for Argos location class 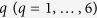 associated with the *i*^th^ observation. Both 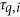 and 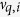 were fixed at the values in Table 1, as per^11^. However, the parameter 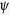 is estimated and used to re-scale the 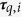
’s thus accounting for potential differences in performance between tags or other individual-level factors that could lead to differences in the scale of the Argos location class errors. This approach renders the fixed 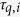
’s, independently estimated from a single dataset^33^, broadly appropriate for other species. Clearly, the estimates can be further refined for particular species or geographic locations if suitable independent error-validation datasets are available. In the hierarchical model, 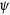 is estimated separately for each individual. The ***μ***_*i*_’s were derived from the estimated location states 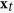 via:


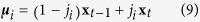


where 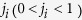 is the proportion of the time step between location states 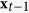 and 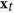 that elapsed prior to the *i*^th^ observation. This approach assumes the seals travel in a straight line between times 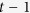 and *t*.

### Analyses

The models were fit to the simulated and Weddell seal data using the JAGS software^35^,^36^ from within R^37^. Models fit to the simulated data had a nominal 1 hr time step, matching the resolution of the simulated data. For the Weddell seals, the models were fit with a 6 hr time step, resulting in a average of two observations per time step. In all cases, two Markov chain Monte Carlo (MCMC) chains of 60 000 samples were run and the first 40 000 from each chain were discarded as a burn-in. Posterior inference was performed from the remaining 20 000 samples per chain after thinning by a factor of 20 to reduce within-chain sample autocorrelation, yielding a final 2 000 samples from the joint posterior. Model convergence was assessed by ascertaining whether posterior samples were stationary, the individual MCMC chains were well-mixed, within-chain sample autocorrelation was relatively low, and the Brooks-Gelman-Rubin potential scale reduction factors 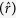
38 were ≤1.1. Initial values for all parameters were drawn randomly and independently for each chain to ensure adequate over-dispersion, an underlying assumption of the 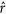 statistic.

Computation times for analysis of the simulated and Weddell seal datasets are provided in Table S2.

## Results

### Simulations

**Figure 1:**
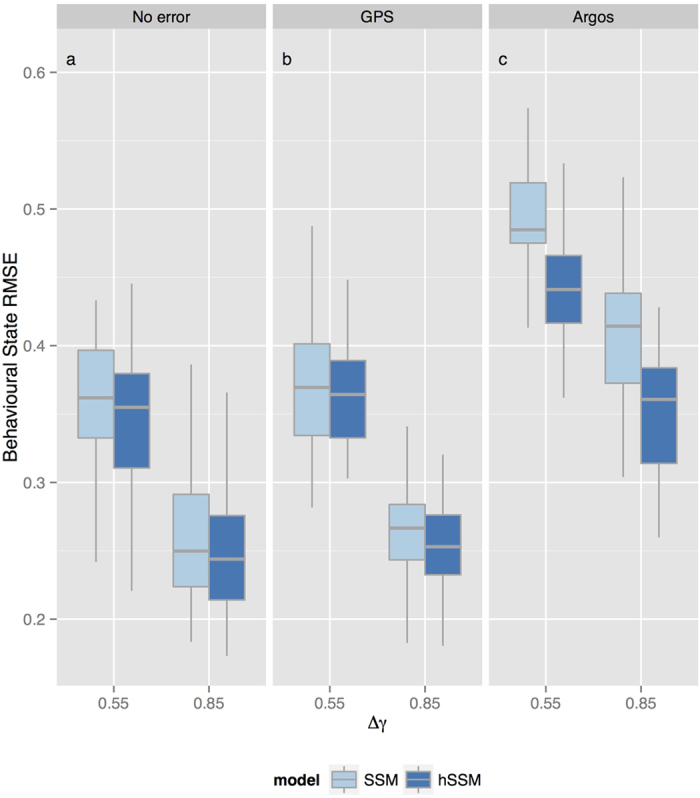
Boxplots of the behavioural state RMSE from a hierarchical state-space model (hSSM) fit simultaneously to simulated tracks and a non-hierarchical state-space model (SSM) fit individually to the same simulated tracks. The RMSE is compared between scenarios with (**a**) locations observed without error, (**b**) locations with typical GPS errors, or (**c**) locations with typical Argos errors, and with a small (0.55) or large (0.85) difference between the move persistence parameters 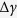 characterising the two behavioural states (see Table 1 for parameter values). Each box displays the distribution of RMSE values from 50 simulations, horizontal bars are medians, boxes give the inter-quartile range, whiskers give the full range.

Behavioural state RMSE was consistently lower when behavioural states were estimated via the hSSM, across all scenario combinations (Fig. 1). The differences in median RMSE between the hSSM and SSM with either small or large 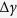, however, was very small under the no error and GPS error scenarios. Despite the consistent improvement in RMSE, the hSSM clearly can not fully compensate for difficulties in estimating behavioural states when movement characteristics differ relatively little between the states - simulated here as 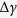. Behavioural state RMSE was consistently lower when 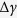 was large, regardless of model or location data type. On average, behavioural state RMSE was highest under the Argos data scenario (Fig. 1b), but this was also where using the hSSM conferred a larger reduction in RMSE relative to the SSM. Combined, these results imply that hSSM's should be preferred to SSM's for behavioural state estimation when fitting to Argos location data.

### Weddell seals

**Figure 2:**
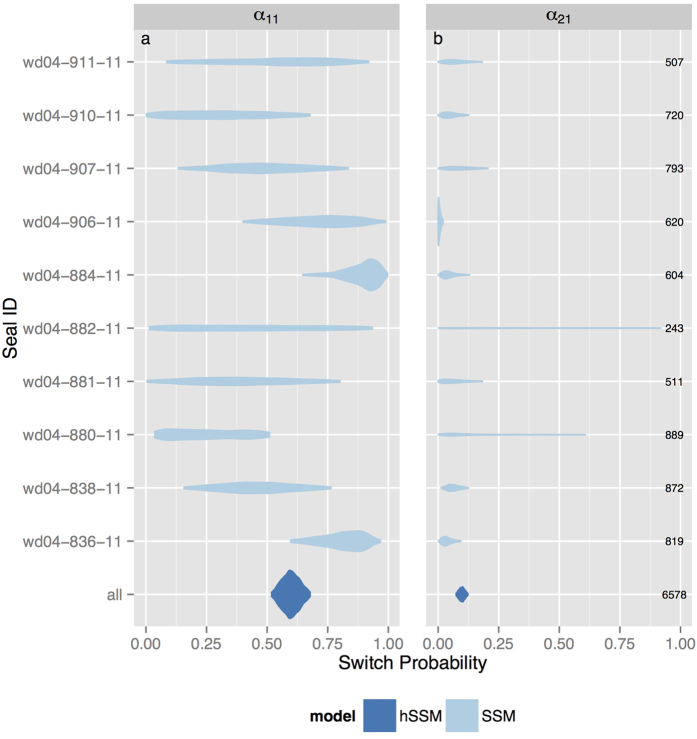
Violin plots of the posterior distributions (95% Highest Posterior Density Interval) for the behavioural state switching probabilities (a) 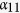 and (b) 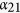. These parameters were estimated separately for each of the 10 Weddell seals via the SSM and across seals via the hSSM. 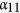 is the probability of remaining in the transient state and 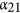 is the probability of switching from the ARS state to the transient state. Track sample sizes are displayed at right in (**b**). Violin heights are scaled to have equal areas within panels.

The fits of the SSM to the Weddell seal data revealed that individual datasets contained relatively little information about 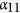, the probability of remaining in the transient state (Fig. 2a; light blue; highest posterior density intervals, HPDI, spanned >50% of possible values for 7 of 10 individuals). The 3 individuals (wd04-836-11, wd04-880-11, wd04-884-11) that had reasonably well defined 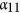 posteriors had relatively large numbers of observed locations, but not necessarily the largest. The 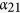 parameter estimates from the SSM, the probability of switching from the ARS state to the transient state, were reasonably well-estimated except for 2 individuals whose 95% HPDI's spanned >50% of possible values (wd04-880-11 and wd04-882-11; Fig. 2b). In contrast, the 95% HPDI's for the 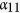 and 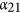 parameters from the hSSM imply considerable information about the switching probabilities when the 10 seal tracks are combined hierarchically (Fig. 2; dark blue).

**Figure 3:**
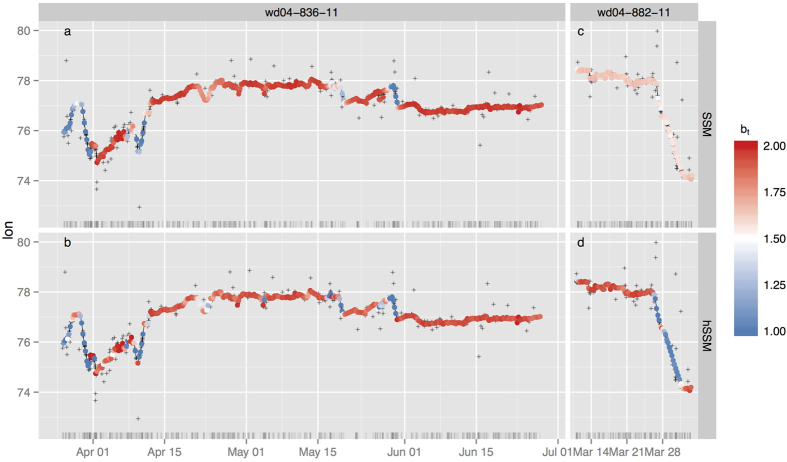
Posterior mean longitude time-series coloured by the posterior mean behavioural state for seals wd04-882-11 (a,b) and wd04-836-11 (c,d). Panels (**a–c**) are estimates from the SSM and panels (**b–d**) are estimates from the hSSM. Longitudes in (**a**,**b**) are shifted −2.5° to lie within the range of (**c**,**d**), allowing better visualisation of behavioural state transitions across both seals. The grey + symbols display the observed longitudes and the rug along the x-axis denotes their time sequence.

The difference in behavioural state estimation between the two models is also noticeable (Fig. 3). For simplicity and clarity of display, I focus on 2 individuals' posterior mean behavioural states along their longitude time-series. See Fig. S2 for all other individuals. The hSSM resolves shorter lasting behavioural transitions that are missed entirely or estimated with greater uncertainty by the SSM (Fig. 3a,b). The hSSM also better resolves entire behavioural state sequences for individuals with less information about behavioural switches (Fig. 3c,d). In each case, the hSSM estimates of transient behaviour (blue circles) sensibly align with relatively rapid changes in longitude, whereas estimates of ARS behaviour align with slower changes in longitude.

## Discussion

Hierarchical state-space models can improve the inference of behavioural states from animal movement data compared to inferences made with non-hierarchical models fit to individual datasets. Analysis of simulated movement paths showed that the advantage conferred by the hSSM was greatest when location error was typical of Argos satellite data. In contrast, when there is little or no error in location data there is little gain in using an hSSM.

Analysis of Argos tracks from Weddell seals showed that the hSSM could better estimate behavioural state switching probabilities from the combined dataset, whereas the SSM in most cases had difficultly identifying these parameters for individual seals. This parameter identifiability issue in turn leads to generally greater uncertainty in behavioural state estimates and partial or complete misidentification of short-lasting behavioural state transitions. In contrast, the hSSM generally had less uncertainty in the behavioural state estimates and was able to identify short periods (i.e. 6–24 h) of relatively rapid movement that the SSM could not.

The relatively poor estimability of the 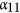 parameter, the probability of remaining in the transient movement state, within the SSM reflects the general movement patterns and ecology of Weddell seals. These seals are an ice obligate species, foraging within the sea ice, requiring fast ice for breeding and moulting, and spending significant time hauled out upon ice^39^,^40^. As a consequence, their movements tend to remain highly localised, dominated by periods of low travel rate and directional persistence, and the magnitude of error in the Argos satellite-derived location data is large relative to this predominantly local movement scale. It is not surprising that the SSM had difficulty in estimating the state switching probabilities and the behavioural states as there is relatively poor contrast in the movements of individual seals. By estimating movement and switching parameters across multiple individual seal datasets, the hSSM has much more information about changes in movement patterns than typically exists in any single Weddell seal dataset. This “borrowing of strength” across multiple datasets is a key advantage of a hierarchical or meta-analytic approach^24^,^41^,^42^ and may be the only viable option for inferring latent behavioural states from animal movement data subject to large location errors relative to the scale of movement.

The hierarchical model used here is simple because it assumes that movement parameters are identical among individuals rather than assume that movement parameters differ among individuals but arise from common distributions^25^,^27^. Despite this simplicity, the model clearly improves inference of latent behavioural states in both the simulated and real datasets. Nevertheless, a fully hierarchical model with movement parameters estimated at both the individual and population levels may yield further improvements and will clearly be essential when inference is desired at both levels. These models can be challenging to implement. In the frequentist case, the number of hierarchical parameters (random effects) must typically be small to avoid prohibitively complex numerical integration of the likelihood over the random effects^14^. In the Bayesian case, MCMC algorithms provide a natural approach to fitting hierarchical models even across relatively large numbers of parameters, but these are computationally slow and frustratingly tricky to reach convergence. The long computation time required for these models, here on the order of 1.25 days to fit the hSSM model to 50 simulated tracks of 200 time steps, is a clear impediment to their routine and broad adoption. However, the hSSM takes approximately the same time to fit as the SSM, so there is no further penalty imposed by taking a hierarchical approach.

Alternative fast approaches, such as approximate Bayesian inference via integrated nested Laplace approximations (INLA)^43^ and automatic differentiation and Laplace approximation via AD Model Builder^44^ or Template Model Builder (TMB; developed by Kasper Kristensen and freely available at http://www.tmb-project.org)^45^, will be essential to further expansion of the process-based behavioural modelling toolbox. Approaches such as these hold promise but have not yet been implemented in the current context.

Using a Bayesian hidden Markov modelling approach and datasets simulated without location error^29^, similarly found poor behavioural state classification accuracy when movement characteristics of the two states were relatively indistinct from one another. However, they also found that classification accuracy approached 100% when movement characteristics were distinct between states and accuracy remained relatively high as long as the behavioural states were present in roughly equal proportions. Here the behavioural state estimates had relatively high RMSE's, except in the GPS scenario when movement characteristics differ greatly between the states. A number of factors may contribute to this apparent discrepancy between studies.

First, both the model and the simulated data in Beyer *et al.*^29^ had no measurement error component. This should to better behavioural state estimation, however, the simulation scenario without measurement error explored here (Fig. S2) implied little difference in behavioural state estimation error to the GPS error scenario (Figs 2 and S2).

Second, the Kappa statistic^34^ used to classify behavioural state accuracy against known simulated states does not take into account uncertainty in the behavioural state estimates. Under some circumstances this could lead to an impression of high classification accuracy but with considerable uncertainty in the actual state estimates, whereas the RMSE statistic used here increases with state estimation uncertainty.

Third, the models used in Beyer *et al.*^29^ differed structurally from those used here. They used (1) a simpler mixture model where, unlike the Markovian state transitions assumed here, the behavioural states were independent of previous states and (2) a more complex patch-based hidden semi-Markov model^14^ where both habitat and time since last behavioural switch determined the current probability of switching between states. Hidden semi-Markov models have been shown to perform better than hidden Markov models in estimating latent behavioural states from GPS tracking data on the movement behaviours of fishing vessels^15^. Gurarie *et al.*^46^ show that a variety of methods, including 2- and 3-state hidden Markov models with correlated random walk processes, all have advantages and potential disadvantages depending on the nature of the movement data analysed. Hidden Markov models were sensitive to autocorrelation in velocity, an effect that will compound with increasingly high temporal resolution data^46^. The analogous SSM/hSSM considered here explicitly accounts for movement autocorrelation via the *γ* parameter and should be less sensitive to this effect, but this will also depend on the chosen time step relative to the temporal resolution of the data. Care needs to be taken to choose an appropriate time step given both the data resolution and the potential behaviours hidden in the data. This highlights the importance of understanding the structure of the movement data prior to attempting to fit behavioural switching models^46^.

The models examined here are simple but necessary abstractions of reality. Animals engage in numerous behaviours across a range of temporal scales, many of which are unrelated to their observable horizontal movements. Behavioural switching models simply classify the coarse aggregate of animals' behaviour at a temporal scale greater than the minimum observed sampling interval based on fundamental differences in movement inferred from the observed locations. These tools are useful in identifying where and when animals engage in different activities (e.g., search, forage, rest, migrate)^8^,^11^,^22^ and relating these to environmental correlates^8^,^10^,^27^ to infer potential effects of environmental change^7^. They can, however, benefit from more realism built into the assumed movement process(es), for example, explicitly accounting for the role of memory^12^,^14^,^47^ and ancillary information about animals' activity, such as diving or resting behaviours^28^. These more realistic movement models will be data-hungry, requiring considerable information about fundamental movement and behavioural parameters. Notwithstanding the need to scale up inferences of movement processes from individuals to populations, hierarchical implementations of these new models may provide the only viable option for proper parameter estimation.

## Additional Information

**How to cite this article**: Jonsen, I. Joint estimation over multiple individuals improves behavioural state inference from animal movement data. *Sci. Rep.* **6**, 20625; doi: 10.1038/srep20625 (2016).

## Supplementary Material

Supplementary Information

## Figures and Tables

**Table 1:** Parameters used to simulate movement paths under the four scenarios.

**Data type**	**Δ*****γ***	***γ***_1_	***γ***_2_	***θ***_**1**_	***θ***_**2**_	***α***_**1**_	***α***_**2**_	**∑ (km)**	**Ω**	***τ***_***q***_	***v***_***q***_
GPS	0.85	0.95	0.10	0	*π*	0.90	0.10	5	0.05	–	–
GPS	0.55	0.65	0.10	0	*π*	0.90	0.10	5	0.05	–	–
Argos	0.85	0.95	0.10	0	*π*	0.90	0.10	5	–	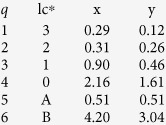	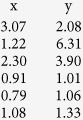
Argos	0.55	0.65	0.10	0	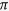	0.90	0.10	5	–		

Values given under **∑** and **Ω** are SD in km and are equal in both x and y directions. For simplicity, covariance terms are set to 0. Parameter estimates for ***τ***_*q*_ and ***v***_*q*_ are from^11^. Scale parameters ***τ***_*q*_ are in km. lc is the Argos location quality class.
