# Supplementary material for: Joint estimation over multiple individuals improves behavioural state inference from animal movement data
Source: Sci Rep. 2016 Feb 8;6:20625. doi: 10.1038/srep20625 (PMC4745009; doi:10.1038/srep20625)
Supplement: Supplementary Information [file srep20625-s1.pdf]

Joint estimation over multiple individuals improves behavioural state inference from animal movement data

Ian Jonsen

Supplementary Information

1 Summary

This Appendix describes: (1) the prior distributions used and general structure of the state-space models; (2) the computation times required for fitting the SSM and hSSM to the simulated and Weddell seal datasets; (3) the R code for simulating movement paths from the behavioural switching state-space model described in the Methods and Materials; (4) the R code used to prepare the simulated data and call JAGS to fit the movement models; (5) the JAGS code for the SSM and hSSM models; (6) the R code used to prepare the Weddell seal data and fit the SSM and hSSM model; (7) the JAGS code for the SSM and hSSM models fit to the Weddell seal data (Note, these models have identical process models as those in (5) but include a regularisation in the observation model to account for temporally irregular observations). The Appendix also includes a figure (Fig. S1) of example simulated paths, results of additional simulations of tracks without observation error (Fig S2), and a multi-page figure (Fig. S3) of SSM- and hSSM-estimated behavioural states for the remaining 8 Weddell seal datasets not presented in the Results (Fig. 3).

2 Prior distributions and general model structure

Table S1: Prior distributions for model parameters.

Parameter	Description	Prior Distribution
θ_1	Mean turn angle (transient)	$\pi(2\ \text{Beta}(10, 10) - 1)$
θ_2	Mean turn angle (ARS)	$2\pi\ \text{Beta}(10, 10)$
γ_1	Move persistence (transient)	Beta(5, 2)
γ_2	Move persistence (ARS)	Beta(2, 5)
α_{11}	Switch probability (remain in transient)	Beta(1, 1)
α_{21}	Switch probability (ARS to transient)	Beta(1, 1)
λ_1	Probability of transient state at t=1	Beta(1, 1)
λ_2	Probability of ARS state at t=1	$1 - \lambda_1$
Σ	Process variance in longitude and latitude	Wishart($\Omega^\dagger$, 2)

$^\dagger\ \Omega = \begin{bmatrix} 1 & 0 \\ 0 & 1 \end{bmatrix}$

$$\begin{aligned}
\text{Priors}: &\quad h(\theta, \gamma, \alpha, \lambda, \boldsymbol{\Sigma}) && \\
\text{Process model}: &\begin{cases} g_1(\mathsf{x}_1, b_1 | y_1, \lambda, \psi^\dagger, \tau^\dagger, \nu^\dagger) & \text{State } t = 1 \\ g_2(\mathsf{x}_2 | \mathsf{x}_1, \boldsymbol{\Sigma}) & \text{State } t = 2 \\ g_t(\mathsf{x}_{t+1}, b_t | \mathsf{x}_t, \mathsf{x}_{t-1}, b_{t-1}, \theta, \gamma, \alpha, \boldsymbol{\Sigma}) & \text{State } t \geq 2 \end{cases} \\
\text{Observation model}: &\quad f_t(\mathsf{y}_t | \mathsf{x}_t, \psi^\dagger, \tau^\dagger, \nu^\dagger) \qquad \text{State } t \geq 2
\end{aligned}$$

† in all cases τ and ν are fixed parameters, see Table 1 for parameter values. ν is only used in the Argos location models, and ψ is only used in the models for Weddell seal (Argos) data.

3 Computation times

All models were fit on a 2014 Mac Pro with 6, 3.5 GHz dual-core processors, 64 Gb RAM and 1 Tb solid-state hard-drive. R version 3.2.2 (Fire Safety) and JAGS v3.4.0 were used.

Table S2: Computation times for the SSM and hSSM fits to the simulated and Weddell seal datasets.

Model	Dataset	Time (h)
SSM	Simulated	27.9
hSSM	Simulated	28.5
SSM	Weddell seal	11.25
hSSM	Weddell seal	12.1

4 R code for simulating movement paths

`simTrack`'s arguments are the number of time steps (T, nominally in hours), the mean turn angles for the two movement states (theta, in radians), the move persistence (gamma), the state switching probabilities (alpha), the 2x2 variance-covariance matrix for the process variability (Sigma). The parameters theta, gamma are vectors of length 2 with values corresponding to the behavioural states ”transient” and ”ARS”, respectively. The parameter alpha is also a vector of length 2, with the first value giving the probability of remaining in the ”transient” state and second giving the probability of switching from the ”ARS” state to the ”transient” state.

```
`simTrack` = function(T = 100, theta = c(0, pi), gamma = c(0.95, 0.1),
  alpha = c(0.9, 0.2), Sigma = matrix(c(5,0,0,5),2,2), err){
require(mvtnorm)
start.date = strptime(format(Sys.time(), "%d/%m/%y %H:%M:%S"),
  "%d/%m/%y %H:%M:%S", tz = "GMT")
Y = X = matrix(NA, T, 2)
TdX = matrix(NA, T-1, 2)
X.mn = matrix(NA, T-1, 2)
b = c()
mu = c()
tau.x = c()
tau.y = c()
nu.x = c()
nu.y = c()
X[1, ] = rmvnorm(1, c(1000,1000), Sigma) #randomize starting position
X[2, ] = rmvnorm(1, X[1,], Sigma)
b[1] = 1
for(i in 2:(T-1)){
  b[i] = sample(1:2, 1, prob=c(alpha[b[i-1]],1-alpha[b[i-1]]), replace=TRUE)
  TdX[i,1] = cos(theta[b[i]]) * (X[i,1] - X[i-1,1]) +
    sin(theta[b[i]]) * (X[i,2] - X[i-1,2])
  TdX[i,2] = -sin(theta[b[i]]) * (X[i,1] - X[i-1,1]) +
    cos(theta[b[i]]) * (X[i,2] - X[i-1,2])
  X.mn[i,] = X[i,] + TdX[i,] * gamma[b[i]]
  X[i+1,] = rmvnorm(1, X.mn[i,], Sigma)
  }
b[T] = sample(1:2, 1, prob=c(alpha[b[T-1]],1-alpha[b[T-1]]), replace=TRUE)
if(err=="gps"){
  tau.x = tau.y = 0.05# SD = 50 m
  nu.x = nu.y = 10000   # Gaussian errors
```

```
  Y[,1] = X[,1] + tau.x * rt(T, nu.x)
  Y[,2] = X[,2] + tau.y * rt(T, nu.y)
  lc = rep("f", dim(X)[1])
  }
else if(err=="argos"){
  ## Randomly draw lc's
  ## Use class proportions from IMOS Southern elephant seal data as
  ## probability vector
  lc = factor(sample(c(3,2,1,0,"A","B"), T, replace=TRUE,
    prob=c(0.03,0.04,0.059,0.145,0.371,0.353)), levels=c(3,2,1,0,"A","B"),
    ordered=TRUE)
  ## Error scale and df's from Jonsen et al. (2005) Ecology (in km)
  tau.x[1] = 0.2898660
  tau.x[2] = 0.3119293
  tau.x[3] = 0.9020423
  tau.x[4] = 2.1625936
  tau.x[5] = 0.5072920
  tau.x[6] = 4.2050261
  tau.y[1] = 0.1220553
  tau.y[2] = 0.2605126
  tau.y[3] = 0.4603374
  tau.y[4] = 1.607056
  tau.y[5] = 0.5105468
  tau.y[6] = 3.041276
  nu.x[1] = 3.070609
  nu.x[2] = 1.220822
  nu.x[3] = 2.298819
  nu.x[4] = 0.9136517
  nu.x[5] = 0.786954
  nu.x[6] = 1.079216
  nu.y[1] = 2.075642
  nu.y[2] = 6.314726
  nu.y[3] = 3.896554
  nu.y[4] = 1.010729
  nu.y[5] = 1.057779
  nu.y[6] = 1.331283
  Y[,1] = X[,1] + tau.x[as.numeric(lc)] * rt(T, nu.x[as.numeric(lc)])
  Y[,2] = X[,2] + tau.y[as.numeric(lc)] * rt(T, nu.y[as.numeric(lc)])
  tau.x = tau.x[as.numeric(lc)]
  tau.y = tau.y[as.numeric(lc)]
  nu.x = nu.x[as.numeric(lc)]
  nu.y = nu.y[as.numeric(lc)]
  }
## time interval is nominally 1 h
dates = seq(start.date, start.date + (T-1) * 3600, by=3600)
simdat = data.frame(date=dates, x=X[,1], y = X[,2], x.obs=Y[,1], y.obs=Y[,2],
  lc, b, theta=theta[b], gamma=gamma[b], sigma=Sigma[1,1],
  tau.x, tau.y, nu.x, nu.y)

simdat
}
```

5 R code for fitting SSM or hSSM models to simulated data

I use a series of nested R functions to prepare the movement data for fitting the SSM and hSSM via JAGS. The outer function `fitSSM` takes the simulated data as input (`indata`) and the arguments are the SSM or hSSM to be fit [SSMs: DCRWS (GPS data) or rDCRWS (Argos data); hSSMs: hDCRWS (GPS data) or hrDCRWS (Argos data)], the time step for the model (1 h in all cases), the settings for MCMC sampling: the burnin and adaptation phase (40,000 samples in all cases), the posterior samples (20,000 in all cases), the degree of thinning (retaining 1 in 20 samples in all cases), and the number of parallel chains (2 in all cases).

```
`fitSSM` = function (indata, model="hrDCRWS", tstep=1/24, adapt=40000,
  samples=20000, thin=20, chains=2)
{
require(rjags)
source("ssm.r")
source("hssm.r")
N = length(unique(indata$id))
T = nrow(indata)/N
dat = lapply(1:N, function(i){
  with(subset(indata, id==unique(id)[i]), list(id=id[1],
    y=cbind(x.obs,y.obs), tau=cbind(tau.x,tau.y),
    nu=cbind(nu.x,nu.y), T=T, first.date=date[1],
    dts=date, tstep=tstep))
  })
if(model %in% c("hDCRWS", "hrDCRWS")){
  fit = hssm(dat, model = model, model.file = model.file, adapt = adapt,
    samples = samples, thin = thin, chains = chains)
  }
else {
  fit = ssm(dat, model = model, model.file = model.file, adapt = adapt,
    samples = samples, thin = thin, chains = chains)
  }
if(model %in% c("DCRWS","rDCRWS")){
  out = lapply(1:length(fit), function(i){
    dat=subset(indata, id==unique(id)[i])
    list(fit=fit[[i]], data=dat)
  })
}
else {
  out = list(fit=fit, data=indata)
  }
out
}
```

The `fitSSM` function calls `ssm` or `hssm`, depending on whether the SSM or hSSM is to be fit. These functions prepare the data for input to JAGS, create initial values, call JAGS with the appropriate SSM or hSSM models, and restructure the output from JAGS.

```
`ssm` = function (indata, model, adapt, samples, thin, chains, ...)
{
if (!model %in% c("DCRWS","rDCRWS")) stop("model not implemented")
ssm1 = function(input) {
  y.dat = input$y
```

```
T = input$T
id = input$id
idat = data.frame(date=input$dts,x=y.dat[,1],y=y.dat[,2])
idts = seq(input$first.date, by=input$tstep*86400, length.out=T)
ix = predict(loess(x~as.numeric(date), idat, span=0.1),
  newdata=data.frame(date=as.numeric(idts)))
iy = predict(loess(y~as.numeric(date), idat, span=0.1),
  newdata=data.frame(date=as.numeric(idts)))
if((sum(is.na(ix))+sum(is.na(iy)))>0){
  ix[is.na(ix)] = ix[which(is.na(ix))-1]
  iy[is.na(iy)] = iy[which(is.na(iy))-1]
}
x.init = cbind(ix,iy)
start.date = input$first.date
dist = c(0,sqrt(diff(x.init[,1])^2+diff(x.init[,2])^2))
dist.ma = filter(dist, rep(1/5,5), sides=2, circular=TRUE)
b.init = rep(1, nrow(x.init))
b.init = ifelse(dist.ma < median(dist.ma), 2, b.init)
jags.data = list(y = y.dat, tau = input$tau, T = T)
if(model == "rDCRWS"){
  jags.data = list(y = y.dat, tau = input$tau, nu=input$nu, T=T)
}
iSigma = matrix(c(1, 0, 0, 1), 2, 2)
jags.inits = list(list(iSigma=iSigma,
  gamma = c(rbeta(1,5,2), rbeta(1,2,5)),
  tmp = c(runif(1,0.45,0.55), runif(1,0,1)),
  alpha = c(rbeta(1,10,2), rbeta(1,2,10)),
  lambda = c(runif(1,0,1), NA), b = b.init, x = x.init),
  list(iSigma=iSigma,
  gamma = c(rbeta(1,5,2), rbeta(1,2,5)),
  tmp = c(runif(1,0.45,0.55), runif(1,0,1)),
  alpha = c(rbeta(1,10,2), rbeta(1,2,10)),
  lambda = c(runif(1,0,1), NA), b = b.init, x = x.init))
jags.params = c("Sigma", "x", "tmp", "theta", "gamma", "lambda",
  "alpha", "b")
model.file = paste(model, ".txt", sep="")
load.module("dic")
burn = jags.model(model.file, jags.data, jags.inits, n.chains=chains,
  n.adapt=adapt/2)
update(burn, n.iter=adapt/2)
psamples = jags.samples(burn, c(jags.params,"deviance","pD"),
  n.iter=samples, thin=thin)
x = apply(psamples$x[,1,,],1, mean)
y = apply(psamples$x[,2,,],1, mean)
x.q = apply(psamples$x[,1,,],1, quantile, c(0.025, 0.5, 0.975))
y.q = apply(psamples$x[,2,,],1, quantile, c(0.025, 0.5, 0.975))
Dbar = summary(psamples$deviance, mean) # posterior mean of the deviance
pD  = summary(psamples$pD, mean) #  the effective number of parameters
DIC = Dbar$stat + pD$stat
b = apply(psamples$b, 1, mean)
b.5 = apply(psamples$b, 1, median)
summary = data.frame(id=as.character(id), date =
  as.POSIXct(input$dts, origin="1970-01-01 00:00:00", tz="GMT"),
```

```
    x, y, x.025=x.q[1,], x.5=x.q[2,], x.975=x.q[3,],
    y.025=y.q[1,], y.5=y.q[2,], y.975=y.q[3,], b, b.5)
  model = model
  step = with(summary, difftime(date[2], date[1], units="hours"))
  out = list(summary=summary, model=model, timestep=step, T=T,
    mcmc=psamples, dic=as.numeric(DIC), dbar=as.numeric(Dbar$stat),
    pd=as.numeric(pD$stat), adapt=adapt, samples=samples, thin=thin,
    chains=chains)
  out
  }
lapply(indata, ssm1)
}
```

The `hSSM` function aggregates individual datasets into a single combined dataset with appropriate indices to allow inference across all individuals simultaneously.

```
`hssm` = function (indata, model, adapt, samples, thin, chains, ...)
{
if (!model %in% c("hDCRWS","hrDCRWS")) stop("model not implemented")
x.init = lapply(indata, function(z){
  idat = data.frame(date=z$dts,x=z$y[,1],y=z$y[,2])
  idts = seq(z$first.date, by=z$tstep*86400, length.out=z$T)
  ix = predict(loess(x~as.numeric(date), idat, span=0.1),
    newdata=data.frame(date=as.numeric(idts)))
  iy = predict(loess(y~as.numeric(date), idat, span=0.1),
    newdata=data.frame(date=as.numeric(idts)))
  cbind(ix,iy)
  })
b.init = lapply(x.init, function(z){
  dist = c(0,sqrt(diff(z[,1])^2+diff(z[,2])^2))
  dist.ma = filter(dist, rep(1/5,5), sides=2, circular=TRUE)
  b = rep(1, nrow(z))
  ifelse(dist.ma < median(dist.ma), 2, b)
  })
x.init = do.call(rbind, x.init)
row.names(x.init) = 1:nrow(x.init)
b.init = as.numeric(unlist(b.init))
N = length(indata)
y.dat = NULL
tau = NULL
nu = NULL
id = NULL
first.date = NULL
dts = NULL
len.y = sapply(indata, function(x) nrow(x$y))
ids = sapply(indata, function(x) x$id)
for(i in 1:N){
  y.dat = rbind(y.dat, indata[[i]]$y)
  tau = rbind(tau, indata[[i]]$tau)
  nu = rbind(nu, indata[[i]]$nu)
  id = c(id, indata[[i]]$id)
  first.date = c(first.date, indata[[i]]$first.date)
  dts = c(dts, indata[[i]]$dts)
```

```
  }
T = indata[[1]]$T
tstep = indata[[1]]$tstep
jags.data = list(y = y.dat, tau = tau, T=T, N=N)
if(model == "hrDCRWS"){
  jags.data = list(y = y.dat, tau = tau, nu=nu, T=T, N=N)
  }
iSigma = matrix(c(1, 0, 0, 1), 2, 2)
jags.inits = list(list(iSigma=iSigma,
  gamma = c(rbeta(1,5,2), rbeta(1,2,5)),
  tmp = c(runif(1,0.45,0.55), runif(1,0,1)),
  alpha = c(rbeta(1,10,2), rbeta(1,2,10)), lambda = c(runif(1,0,1), NA),
  b = b.init, x = x.init), list(iSigma=iSigma,
  gamma = c(rbeta(1,5,2), rbeta(1,2,5)),
  tmp = c(runif(1,0.45,0.55), runif(1,0,1)),
  alpha = c(rbeta(1,10,2), rbeta(1,2,10)), lambda = c(runif(1,0,1), NA),
  b = b.init, x = x.init))
jags.params = c("Sigma", "x", "tmp", "theta", "gamma", "lambda",
  "alpha", "b")
model.file = paste(model, ".txt", sep="")
load.module("dic")
burn = jags.model(model.file, jags.data, jags.inits, n.chains=chains,
  n.adapt=adapt/2)
update(burn, n.iter=adapt/2)
psamples = jags.samples(burn, c(jags.params,"deviance","pD"),
  n.iter=samples, thin=thin)
x = apply(psamples$x[,1,,],1,mean)
y = apply(psamples$x[,2,,],1,mean)
x.q = apply(psamples$x[,1,,],1, quantile, c(0.025, 0.5, 0.975))
y.q = apply(psamples$x[,2,,],1, quantile, c(0.025, 0.5, 0.975))
Dbar = summary(psamples$deviance, mean) # posterior mean of the deviance
pD  = summary(psamples$pD, mean) #  the effective number of parameters
DIC = Dbar$stat + pD$stat
b = apply(psamples$b,1,mean)
b.5 = apply(psamples$b,1,median)
summary = data.frame(id=rep(as.numeric(as.character(id)), each=T),
  date = as.POSIXct(dts, origin="1970-01-01 00:00:00", tz="GMT"),
  x, y, x.025=x.q[1,], x.5=x.q[2,], x.975=x.q[3,], y.025=y.q[1,],
  y.5=y.q[2,], y.975=y.q[3,], b, b.5)
model = model
step = with(summary, difftime(date[2], date[1], units="hours"))
out = list(summary=summary, model=model, timestep=step, data=y.dat,
  T=T, N=N, mcmc=psamples, dic=as.numeric(DIC),
  dbar=as.numeric(Dbar$stat), pd=as.numeric(pD$stat), adapt=adapt,
  samples=samples, thin=thin, chains=chains)
out
}
```

These R functions can be called conveniently via a single script, ensuring that both the hSSM and SSM models are fit to the same simulated datasets. The following example is for the Argos data scenario with large $\Delta\gamma$ (see Table 1):

```
## hierarchical vs non-hierarchical fits - Argos errors
```

```
source("simTrack.R")
source("fitSSM.R")
N = 50
T = 200
theta = c(0, pi)
gamma = c(0.95, 0.1)
alpha = c(0.9, 0.2)
vcov = matrix(c(5,0,0,5), 2, 2)
err = "argos"
tstep = 1/24
adapt = 40000
samples = 20000
thin = 20
simdat = lapply(1:N, function(i) simTrack(T, theta, gamma, alpha,
  vcov, err))
simdat = do.call(rbind, simdat)
indata = data.frame(id=rep(1:N, each=T), simdat)
hfit = fitSSM(indata, model="hrDCRWS", tstep, adapt, samples, thin)
ifit = fitSSM(indata, model="rDCRWS", tstep, adapt, samples, thin)
```

6 JAGS code: non-hierarchical (SSM) switching model for simulated GPS data

```
## JAGS code for DCRWS.txt model: non-hierarchical with Gaussian obs'n model
## data block with constants and hyperparameters for state var-cov matrix
data {
pi <- 3.141592653589
Omega[1,1] <- 1
Omega[1,2] <- 0
Omega[2,1] <- 0
Omega[2,2] <- 1

## Initial state location set equal to the first observed location
first.loc[1] <- y[1,1]
first.loc[2] <- y[1,2]
}
## model block with priors, state process model, and observation model
model {
## ----------------------- Prior distributions -----------------------
## state process var-covar matrix, Sigma
iSigma[1:2,1:2] ~ dwish(Omega[,], 2)
Sigma[1:2,1:2] <- inverse(iSigma[,])

## mean turn angle: theta[1] (transient), theta[2] (ARS)
tmp[1] ~ dbeta(10, 10)
tmp[2] ~ dbeta(10, 10)
theta[1] <- (2 * tmp[1] - 1) * pi
theta[2] <- (tmp[2] * pi * 2)

## move persistence: gamma[1] (transient), gamma[2] (ARS)
gamma[1] ~ dbeta(5, 2)
gamma[2] ~ dbeta(2, 5)

## behavioural state switching probabilities: alpha ``matrix''
alpha[1] ~ dbeta(1, 1)
alpha[2] ~ dbeta(1, 1)

## probabilities of initial behavioural state (transient or ARS)
lambda[1] ~ dbeta(1, 1)
lambda[2] <- 1 - lambda[1]
## -----------------------------------------------------------------
## ---------------------- State Process Model ----------------------
## randomly specify initial behavioural state, b[1]
b[1] ~ dcat(lambda[])

## randomly specify location of first state, x[1,1:2]
x[1,1] ~ dnorm(first.loc[1], tau[1,1]^-2)
x[1,2] ~ dnorm(first.loc[2], tau[1,2]^-2)

## randomly specify location of second state, x[2,1:2]
x[2,1:2] ~ dmnorm(x[1,], iSigma[,])
```

```
## Loop over the 2 to T-1 time steps
for(t in 2:(T-1)){
  ## randomly specify the time t behavioural state, b[t]
  phi[t,1] <- alpha[b[t-1]]
  phi[t,2] <- 1 - alpha[b[t-1]]
  b[t] ~ dcat(phi[t,])

  ## randomly specify the time t+1 location state, x[t+1,1:2]
  x.mn[t,1] <- x[t,1] + (cos(theta[b[t]]) * (x[t,1] - x[t-1,1]) -
    sin(theta[b[t]]) * (x[t,2] - x[t-1,2])) * gamma[b[t]]
  x.mn[t,2] <- x[t,2] + (sin(theta[b[t]]) * (x[t,1] - x[t-1,1]) +
    cos(theta[b[t]]) * (x[t,2] - x[t-1,2])) * gamma[b[t]]
  x[t+1,1:2] ~ dmnorm(x.mn[t,], iSigma[,])
  }
## randomly specify the last behavioural state, b[T]
zeta[1] <- alpha[b[T-1]]
zeta[2] <- 1 - zeta[1]
b[T] ~ dcat(zeta[])
## ------------------------------------------------------------------
## ----------------------- Observation Model -----------------------
for(t in 2:T){
  y[t,1] ~ dnorm(x[t,1], tau[t,1]^-2)
  y[t,2] ~ dnorm(x[t,2], tau[t,2]^-2)
  }
}
```

7 JAGS code: non-hierarchical (SSM) switching model for simulated Argos data

```
## JAGS code for rDCRWS.txt model: non-hierarchical with t-distribution
##     obs'n model
## data block with constants and hyperparameters for state var-cov matrix
data {
pi <- 3.141592653589
Omega[1,1] <- 1
Omega[1,2] <- 0
Omega[2,1] <- 0
Omega[2,2] <- 1

## Initial state location set equal to the first observed location
first.loc[1] <- y[1,1]
first.loc[2] <- y[1,2]
}
## model block with priors, state process model, and observation model
model {
## ----------------------- Prior distributions -----------------------
## state process var-covar matrix, Sigma
iSigma[1:2,1:2] ~ dwish(Omega[,], 2)
Sigma[1:2,1:2] <- inverse(iSigma[,])

## mean turn angle: theta[1] (transient), theta[2] (ARS)
tmp[1] ~ dbeta(10, 10)
```

```
tmp[2] ~ dbeta(10, 10)
theta[1] <- (2 * tmp[1] - 1) * pi
theta[2] <- (tmp[2] * pi * 2)

## move persistence: gamma[1] (transient), gamma[2] (ARS)
gamma[1] ~ dbeta(5, 2)
gamma[2] ~ dbeta(2, 5)

## behavioural state switching probabilities: alpha ``matrix''
alpha[1] ~ dbeta(1, 1)
alpha[2] ~ dbeta(1, 1)

## probabilities of initial behavioural state (transient or ARS)
lambda[1] ~ dbeta(1, 1)
lambda[2] <- 1 - lambda[1]
## ---------------------------------------------------------------------
## ---------------------- State Process Model ----------------------
## randomly specify initial behavioural state, b[1]
b[1] ~ dcat(lambda[])

## randomly specify location of first state, x[1,1:2]
x[1,1] ~ dt(first.loc[1], tau[1,1]^-2, nu[1,1])
x[1,2] ~ dt(first.loc[2], tau[1,2]^-2, nu[1,2])

## randomly specify location of second state, x[2,1:2]
x[2,1:2] ~ dmnorm(x[1,], iSigma[,])

## Loop over the 2 to T-1 time steps
for(t in 2:(T-1)){
  ## randomly specify the time t behavioural state, b[t]
  phi[t,1] <- alpha[b[t-1]]
  phi[t,2] <- 1 - alpha[b[t-1]]
  b[t] ~ dcat(phi[t,])

  ## randomly specify the time t+1 location state, x[t+1,1:2]
  x.mn[t,1] <- x[t,1] + (cos(theta[b[t]]) * (x[t,1] - x[t-1,1]) -
    sin(theta[b[t]]) * (x[t,2] - x[t-1,2])) * gamma[b[t]]
  x.mn[t,2] <- x[t,2] + (sin(theta[b[t]]) * (x[t,1] - x[t-1,1]) +
    cos(theta[b[t]]) * (x[t,2] - x[t-1,2])) * gamma[b[t]]
  x[t+1,1:2] ~ dmnorm(x.mn[t,], iSigma[,])
  }
## randomly specify the last behavioural state, b[T]
zeta[1] <- alpha[b[T-1]]
zeta[2] <- 1 - zeta[1]
b[T] ~ dcat(zeta[])
## ---------------------------------------------------------------------
## ----------------------- Observation Model -----------------------
for(t in 2:T){
  y[t,1] ~ dt(x[t,1], tau[t,1]^-2, nu[t,1])
  y[t,2] ~ dt(x[t,2], tau[t,2]^-2, nu[t,2])
  }
}
```

8 JAGS code: hierarchical (hSSM) switching model for simulated GPS data

```
## JAGS code for hDCRWS.txt model: hierarchical with Gaussian obs'n model
## data block with constants and hyperparameters for state var-cov matrix
data {
pi <- 3.141592653589
Omega[1,1] <- 1
Omega[1,2] <- 0
Omega[2,1] <- 0
Omega[2,2] <- 1

## Initial state location for N animals set equal to their respective first
##     observed location
for(k in 1:N){
  first.loc[k,1] <- y[T*(k-1)+1,1]
  first.loc[k,2] <- y[T*(k-1)+1,2]
  }
}
## model block with priors, state process model, and observation model
model {
## ----------------------- Prior distributions -----------------------
## state process var-covar matrix, Sigma
iSigma[1:2,1:2] ~ dwish(Omega[,], 2)
Sigma[1:2,1:2] <- inverse(iSigma[,])

## mean turn angle: theta[1] (transient), theta[2] (ARS)
tmp[1] ~ dbeta(10, 10)
tmp[2] ~ dbeta(10, 10)
theta[1] <- (2 * tmp[1] - 1) * pi
theta[2] <- (tmp[2] * pi * 2)

## move persistence: gamma[1] (transient), gamma[2] (ARS)
gamma[1] ~ dbeta(5, 2)
gamma[2] ~ dbeta(2, 5)

## behavioural state switching probabilities: alpha ``matrix''
alpha[1] ~ dbeta(1, 1)
alpha[2] ~ dbeta(1, 1)

## probabilities of initial behavioural state (transient or ARS)
lambda[1] ~ dbeta(1, 1)
lambda[2] <- 1 - lambda[1]

## Loop over the N animals
for(k in 1:N){
  ## ---------------------- State Process Model ----------------------
  ## randomly specify initial behavioural state, b[1] for animal k
  b[T*(k-1)+1] ~ dcat(lambda[])

  ## randomly specify location of first state, x[1,1:2] for animal k
  x[T*(k-1)+1,1] ~ dnorm(first.loc[k,1], tau[T*(k-1)+1,1]^-2)
```

```
  x[T*(k-1)+1,2] ~ dnorm(first.loc[k,2], tau[T*(k-1)+1,2]^-2)

  ## randomly specify location of second state, x[2,1:2] for animal k
  x[T*(k-1)+2,1:2] ~ dmnorm(x[T*(k-1)+1,], iSigma[,])

  ## Loop over the 2 to T-1 time steps for animal k
  for(t in (T*(k-1)+2):(T*k-1)){
    ## randomly specify the time t behavioural state, b[t] for animal k
    phi[t,1] <- alpha[b[t-1]]
    phi[t,2] <- 1 - alpha[b[t-1]]
    b[t] ~ dcat(phi[t,])

    ## randomly specify the time t+1 location state, x[t+1,1:2] for animal k
    x.mn[t,1] <- x[t,1] + (cos(theta[b[t]]) * (x[t,1] - x[t-1,1]) -
      sin(theta[b[t]]) * (x[t,2] - x[t-1,2])) * gamma[b[t]]
    x.mn[t,2] <- x[t,2] + (sin(theta[b[t]]) * (x[t,1] - x[t-1,1]) +
      cos(theta[b[t]]) * (x[t,2] - x[t-1,2])) * gamma[b[t]]
    x[t+1,1:2] ~ dmnorm(x.mn[t,], iSigma[,])
    }
  ## randomly specify the last behavioural state, b[T] for animal k
  zeta[k,1] <- alpha[b[T*k-1]]
  zeta[k,2] <- 1 - zeta[k,1]
  b[T*k] ~ dcat(zeta[k,])
  ## -----------------------------------------------------------------
  ## ----------------------- Observation Model -----------------------
  for(t in (T*(k-1)+2):(T*k)){
    y[t,1] ~ dnorm(x[t,1], tau[t,1]^-2)
    y[t,2] ~ dnorm(x[t,2], tau[t,2]^-2)
    }
  }
}
```

9 JAGS code: hierarchical (hSSM) switching model for simulated Argos data

```
## JAGS code for hrDCRWS.txt model: hierarchical with t-distribution
##     obs'n model
## data block with constants and hyperparameters for state var-cov matrix
data {
pi <- 3.141592653589
Omega[1,1] <- 1
Omega[1,2] <- 0
Omega[2,1] <- 0
Omega[2,2] <- 1

## Initial state location for N animals set equal to their respective first
##     observed location
for(k in 1:N){
  first.loc[k,1] <- y[T*(k-1)+1,1]
  first.loc[k,2] <- y[T*(k-1)+1,2]
  }
}
```

```
## model block with priors, state process model, and observation model
model {
## ----------------------- Prior distributions -----------------------
## state process var-covar matrix, Sigma
iSigma[1:2,1:2] ~ dwish(Omega[,], 2)
Sigma[1:2,1:2] <- inverse(iSigma[,])

## mean turn angle: theta[1] (transient), theta[2] (ARS)
tmp[1] ~ dbeta(10, 10)
tmp[2] ~ dbeta(10, 10)
theta[1] <- (2 * tmp[1] - 1) * pi
theta[2] <- (tmp[2] * pi * 2)

## move persistence: gamma[1] (transient), gamma[2] (ARS)
gamma[1] ~ dbeta(5, 2)
gamma[2] ~ dbeta(2, 5)

## behavioural state switching probabilities: alpha ``matrix''
alpha[1] ~ dbeta(1, 1)
alpha[2] ~ dbeta(1, 1)

## probabilities of initial behavioural state (transient or ARS)
lambda[1] ~ dbeta(1, 1)
lambda[2] <- 1 - lambda[1]

## Loop over the N animals
for(k in 1:N){
  ## ---------------------- State Process Model ----------------------
  ## randomly specify initial behavioural state, b[1] for animal k
  b[T*(k-1)+1] ~ dcat(lambda[])


  ## randomly specify location of first state, x[1,1:2] for animal k
  x[T*(k-1)+1,1] ~ dt(first.loc[k,1], tau[T*(k-1)+1,1]^-2, nu[T*(k-1)+1,1])
  x[T*(k-1)+1,2] ~ dt(first.loc[k,2], tau[T*(k-1)+1,2]^-2, nu[T*(k-1)+1,2])

  ## randomly specify location of second state, x[2,1:2] for animal k
  x[T*(k-1)+2,1:2] ~ dmnorm(x[T*(k-1)+1,], iSigma[,])

  ## Loop over the 2 to T-1 time steps for animal k
  for(t in (T*(k-1)+2):(T*k-1)){
    ## randomly specify the time t behavioural state, b[t] for animal k
    phi[t,1] <- alpha[b[t-1]]
    phi[t,2] <- 1 - alpha[b[t-1]]
    b[t] ~ dcat(phi[t,])

    ## randomly specify the time t+1 location state, x[t+1,1:2] for animal k
    x.mn[t,1] <- x[t,1] + (cos(theta[b[t]]) * (x[t,1] - x[t-1,1]) -
      sin(theta[b[t]]) * (x[t,2] - x[t-1,2])) * gamma[b[t]]
    x.mn[t,2] <- x[t,2] + (sin(theta[b[t]]) * (x[t,1] - x[t-1,1]) +
      cos(theta[b[t]]) * (x[t,2] - x[t-1,2])) * gamma[b[t]]
    x[t+1,1:2] ~ dmnorm(x.mn[t,], iSigma[,])
    }
```

```
    ## randomly specify the last behavioural state, b[T] for animal k
    zeta[k,1] <- alpha[b[T*k-1]]
    zeta[k,2] <- 1 - zeta[k,1]
    b[T*k] ~ dcat(zeta[k,])
    ## -----------------------------------------------------------------
    ## ----------------------- Observation Model -----------------------
    for(t in (T*(k-1)+2):(T*k)){
      y[t,1] ~ dt(x[t,1], tau[t,1]^-2, nu[t,1])
      y[t,2] ~ dt(x[t,2], tau[t,2]^-2, nu[t,2])
      }
    }
  }
```

10 R code for fitting SSM or hSSM models to Weddell seal (Argos) data

```
require(bsam) ## available at http://web.science.mq.edu.au/~ijonsen/code

## IMOS 2011 Weddell seal Argos data - Davis deployments
wese = read.csv("wese.csv", stringsAsFactors=FALSE)

wese$date = with(wese, as.POSIXct(paste(paste(year,month,day,sep="-"),
    time, sep=" "), "\%Y-\%m-\%d \%H:\%M:\%S", tz="GMT"))
wese = with(wese, data.frame(id=individual_id, date=date,
    lc=location_quality, lon=decimal_longitude, lat=decimal_latitude))

## strip out duplicate dates
wese = split(wese, wese$id)
wese = lapply(wese, function(x){
    x = x[order(x$date),]
    x[!diff(x$date)==0,]
    })
wese = do.call(rbind, wese)
wese$id = as.character(wese$id)

## select tracks with a range of sample sizes
ids = unique(wese$id)[c(1:5,7,13:14,17,18)]
wese = subset(wese, id \%in\% ids)
wese = split(wese, wese$id)

## trim out gappy ends of a few tracks
wese[[4]] = subset(wese[[4]], format(date,"\%m") < "06")
wese[[6]] = subset(wese[[6]], format(date,"\%m-\%d") < "05-20")
wese = do.call(rbind, wese)
row.names(wese) = 1:nrow(wese)
wese$lc = as.character(wese$lc)

## fit hSSSM with 6-h timestep, a 40000 sample burnin, 20000 posterior samples
##    from each of 2 chains, retaining every 20th sample for a total of
##    2000 final posterior samples
wese.hsssm = fitSSM(wese, model="hDCRWS", tstep=6/24, adapt=40000,
    samples=20000, thin=20)
```

```
## fit SSSM with 6-h timestep, a 40000 sample burnin, 20000 posterior samples
##    from each of 2 chains, retaining every 20th sample for a total of
##    2000 final posterior samples
wese.sssm = fitSSM(wese, model="DCRWS", tstep=6/24, adapt=40000,
    samples=20000, thin=20)
```

11 JAGS code: non-hierarchical (SSM) switching model for Weddell seal (Argos) data

```
##  "DCRWS" model from Ian D Jonsen, Joanna Mills Flemming and Ransom A Myers
##    (2015) Robust state-space modeling of animal movement data.
##    Ecology 86:2874-2880
##    ian.jonsen@mq.edu.au
## non-hierarchical model with regularisation in obs'n model for
##   Weddell seal (Argos) data

## data block with constants and hyperparameters for state var-cov matrix
data {
  pi <- 3.141592653589

  Omega[1,1] <- 1
  Omega[1,2] <- 0
  Omega[2,1] <- 0
  Omega[2,2] <- 1

  ## Initial state locations set equal to first observed location
  first.loc[1] <- y[1,1]
  first.loc[2] <- y[1,2]
  }
## model block with priors, state process model and observation model
model {
## Prior distributions
## state process var-covar matrix, Sigma
iSigma[1:2,1:2] ~ dwish(Omega[,], 2)
Sigma[1:2,1:2] <- inverse(iSigma[,])

## mean turn angle: theta[1] (transient), theta[2] (ARS)
tmp[1] ~ dbeta(10, 10)
tmp[2] ~ dbeta(10, 10)
theta[1] <- (2 * tmp[1] - 1) * pi
theta[2] <- tmp[2] * pi * 2

## move persistence: gamma[1] (transient), gamma[2] (ARS)
##   dev multiplier ensures gamma[1] > gamma[2], prevents state label switching
gamma[1] ~ dbeta(5, 2)
dev ~ dbeta(1, 1)
gamma[2] <- gamma[1] * dev

## behavioural state switching probabilities: alpha ``matrix''
alpha[1] ~ dbeta(1, 1)
alpha[2] ~ dbeta(1, 1)
```

```
## probabilities of initial behavioural state (transient or ARS)
lambda[1] ~ dunif(0, 1)
lambda[2] <- 1 - lambda[1]

## inflation/deflation parameter for t-distribution scale parameters
logpsi ~ dunif(-10, 10)
psi <- exp(logpsi)

## State process model
## randomly specify initial behavioural state, b[1]
b[1] ~ dcat(lambda[])

## randomly specify location of first state, x[1,1:2]
x[1,1] ~ dt(first.loc[1], itau2[1,1] * psi, nu[1,1])
x[1,2] ~ dt(first.loc[2], itau2[1,2] * psi, nu[1,2])

## randomly specify location of second state, x[2,1:2]
x[2,1:2] ~ dmnorm(x[1,], iSigma[,])

## Loop over the 2 to RegN-1 time steps
for(t in 2:(RegN-1)){
  ## randomly specify the time t behavioural state, b[t]
  phi[t,1] <- alpha[b[t-1]]
  phi[t,2] <- 1 - alpha[b[t-1]]
  b[t] ~ dcat(phi[t,])

  ## randomly specify the time t+1 location state, x[t+1,1:2]
  x.mn[t,1] <- x[t,1] + (cos(theta[b[t]]) * (x[t,1] - x[t-1,1]) -
    sin(theta[b[t]]) * (x[t,2] - x[t-1,2])) * gamma[b[t]]
  x.mn[t,2] <- x[t,2] + (sin(theta[b[t]]) * (x[t,1] - x[t-1,1]) +
    cos(theta[b[t]]) * (x[t,2] - x[t-1,2])) * gamma[b[t]]
  x[t+1,1:2] ~ dmnorm(x.mn[t,], iSigma[,])
}

## randomly specify the time RegN-1 behavioural state, b[RegN-1]
zeta[1] <- alpha[b[RegN-1]]
zeta[2] <- 1 - zeta[1]
b[RegN] ~ dcat(zeta[])

##  Observation model
for(t in 2:RegN){
  for(i in idx[t-1]:(idx[t]-1)){
    ## regularisation via linear interpolation between x[t,1:2] and x[t-1,1:2]
    mu[i,1] <- (1-j[i]) * x[t-1,1] + j[i] * x[t,1]
    mu[i,2] <- (1-j[i]) * x[t-1,2] + j[i] * x[t,2]
    y[i,1] ~ dt(mu[i,1], itau2[i,1] * psi, nu[i,1])
    y[i,2] ~ dt(mu[i,2], itau2[i,2] * psi, nu[i,2])
    }
  }
}
```

12 JAGS code: hierarchical (hSSM) switching model for Weddell seal (Argos) data

```
##  "hDCRWS" model from Ian D Jonsen, Joanna Mills Flemming and Ransom A Myers
##    (2015) Robust state-space modeling of animal movement data.
##    Ecology 86:2874-2880
##    ian.jonsen@mq.edu.au
## hierarchical model with regularisation in obs'n model for
##   Weddell seal (Argos) data

## data block with constants and hyperparameters for state var-cov matrix
data {
  pi <- 3.141592653589

  Omega[1,1] <- 1
  Omega[1,2] <- 0
  Omega[2,1] <- 0
  Omega[2,2] <- 1

  ## Initial state location for N animals set equal to their respective first
  ##   observed location
  for(k in 1:N){
    first.loc[k,1] <- y[Yidx[k],1]
    first.loc[k,2] <- y[Yidx[k],2]
    }
}
## model block with priors, state process model, observation model
model {
## Prior distributions
## state process var-covar matrix, Sigma
iSigma[1:2,1:2] ~ dwish(Omega[,], 2)
Sigma[1:2,1:2] <- inverse(iSigma[,])

## mean turn angle: theta[1] (transient), theta[2] (ARS)
tmp[1] ~ dbeta(10, 10)
tmp[2] ~ dbeta(10, 10)
theta[1] <- (2 * tmp[1] - 1) * pi
theta[2] <- tmp[2] * pi * 2

## move persistence: gamma[1] (transient), gamma[2] (ARS)
##   dev multiplier ensures gamma[1] > gamma[2], prevents state label switching
gamma[1] ~ dbeta(5, 2)
dev ~ dbeta(1, 1)
gamma[2] <- gamma[1] * dev

## behavioural state switching probabilities: alpha ``matrix''
alpha[1] ~ dbeta(1, 1)
alpha[2] ~ dbeta(1, 1)

## probabilities of initial behavioural state (transient or ARS)
lambda[1] ~ dbeta(1, 1)
lambda[2] <- 1 - lambda[1]
```

```
## Loop over N animals
for(k in 1:N){
  ## inflation/deflation parameter for t-distribution scale parameters
  logpsi[k] ~ dunif(-10, 10)
  psi[k] <- exp(logpsi[k])

  ## State process model
  ## randomly specify initial behavioural state, b[1]
  b[Xidx[k]] ~ dcat(lambda[])

  ## randomly specify location of first state
  x[Xidx[k],1] ~ dt(first.loc[k,1], itau2[Xidx[k],1] * psi[k], nu[Xidx[k],1])
  x[Xidx[k],2] ~ dt(first.loc[k,2], itau2[Xidx[k],2] * psi[k], nu[Xidx[k],2])

 ## randomly specify location of second state
  x[(Xidx[k]+1),1:2] ~ dmnorm(x[Xidx[k],], iSigma[,])

  ## Loop over the 2 to RegN-1 time steps (encoded in Xidx for each animal)
  for(t in (Xidx[k]+1):(Xidx[k+1]-2)){
    ## randomly specify the time t behavioural state, b[t]
    phi[t,1] <- alpha[b[t-1]]
    phi[t,2] <- 1 - alpha[b[t-1]]
    b[t] ~ dcat(phi[t,])

    ## randomly specify the time t+1 location state, x[t+1,1:2]
    x.mn[t,1] <- x[t,1] + (cos(theta[b[t]]) * (x[t,1] - x[t-1,1]) -
      sin(theta[b[t]]) * (x[t,2] - x[t-1,2])) * gamma[b[t]]
    x.mn[t,2] <- x[t,2] + (sin(theta[b[t]]) * (x[t,1] - x[t-1,1]) +
      cos(theta[b[t]]) * (x[t,2] - x[t-1,2])) * gamma[b[t]]
    x[t+1,1:2] ~ dmnorm(x.mn[t,], iSigma[,])
    }

  ## randomly specify the time RegN-1 behavioural state, b[RegN-1]
  ##   RegN-1 is indexed by Xidx for each animal
  zeta[k,1] <- alpha[b[Xidx[k+1]-2]]
  zeta[k,2] <- 1 - zeta[k,1]
  b[Xidx[k+1]-1] ~ dcat(zeta[k,])

  ## Observation model
  for(t in (Xidx[k]+1):(Xidx[k+1]-1)){
    for(i in idx[t]:(idx[t+1]-1)){
      ## regularisation via linear interpolation between x[t,1:2] and x[t-1,1:2]
      mu[i,1] <- (1-j[i]) * x[t-1,1] + j[i] * x[t,1]
      mu[i,2] <- (1-j[i]) * x[t-1,2] + j[i] * x[t,2]
      y[i,1] ~ dt(mu[i,q], itau2[i,1] * psi[k], nu[i,1])
      y[i,2] ~ dt(mu[i,q], itau2[i,2] * psi[k], nu[i,2])
      }
    }
  }
}
```

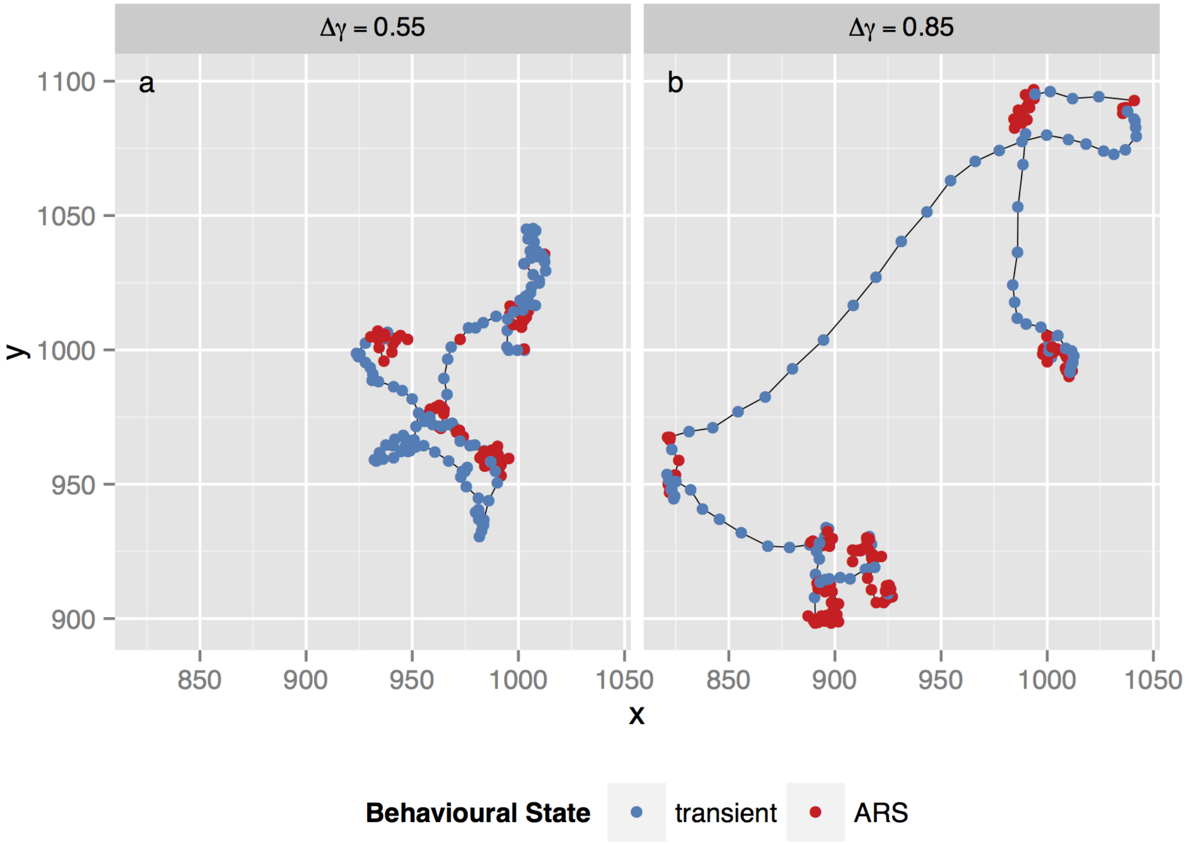

Figure S1: Example simulated paths with (a) small and (b) large differences in move persistence parameters ($\Delta\gamma$) that characterise the transient and ARS behavioural states. Note the lower directional persistence and generally smaller displacements during the transient state in (a) relative to (b).

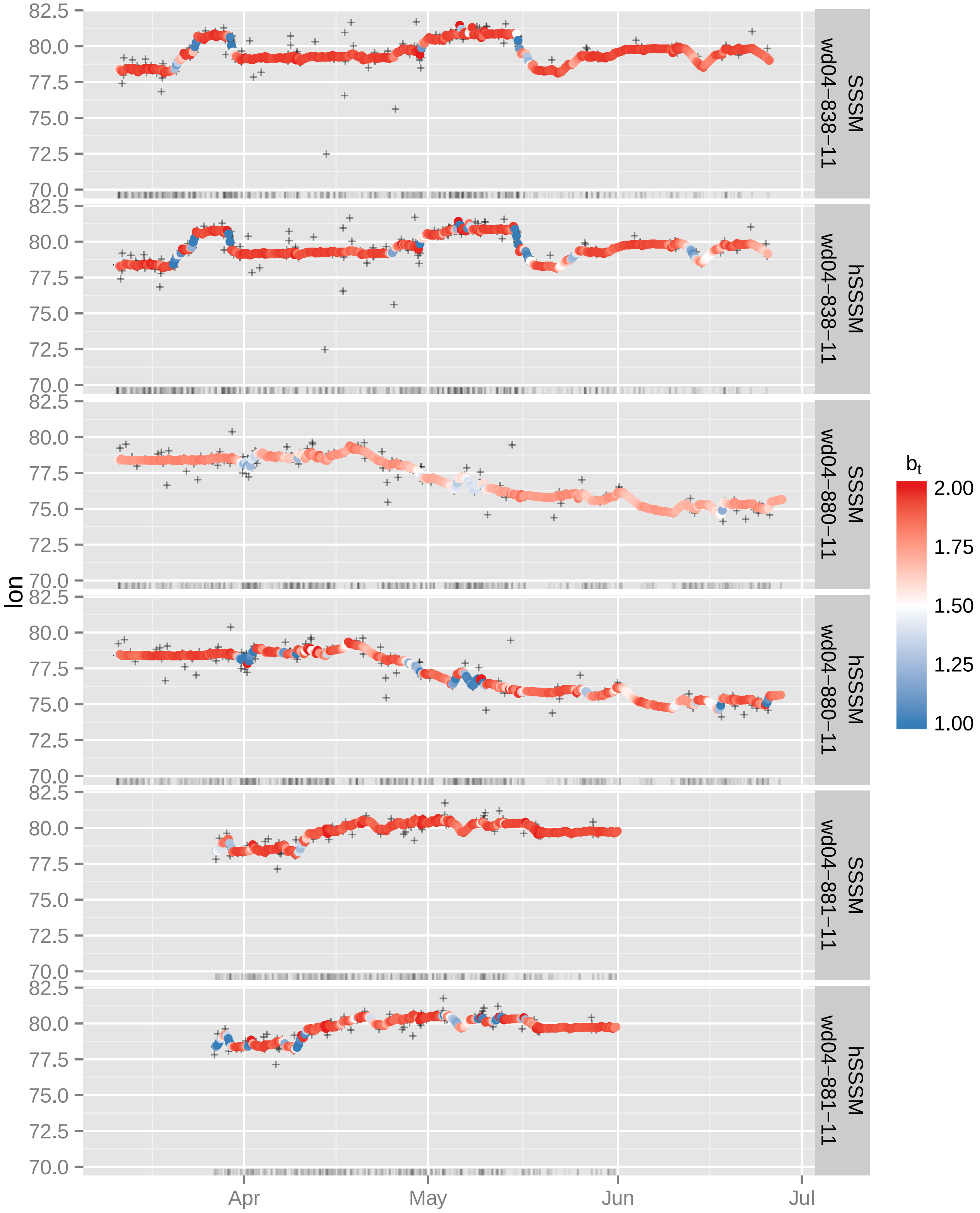

Figure S2: Posterior mean longitude time-series coloured by the posterior mean behavioural state for the 8 seals not included in Fig. 3

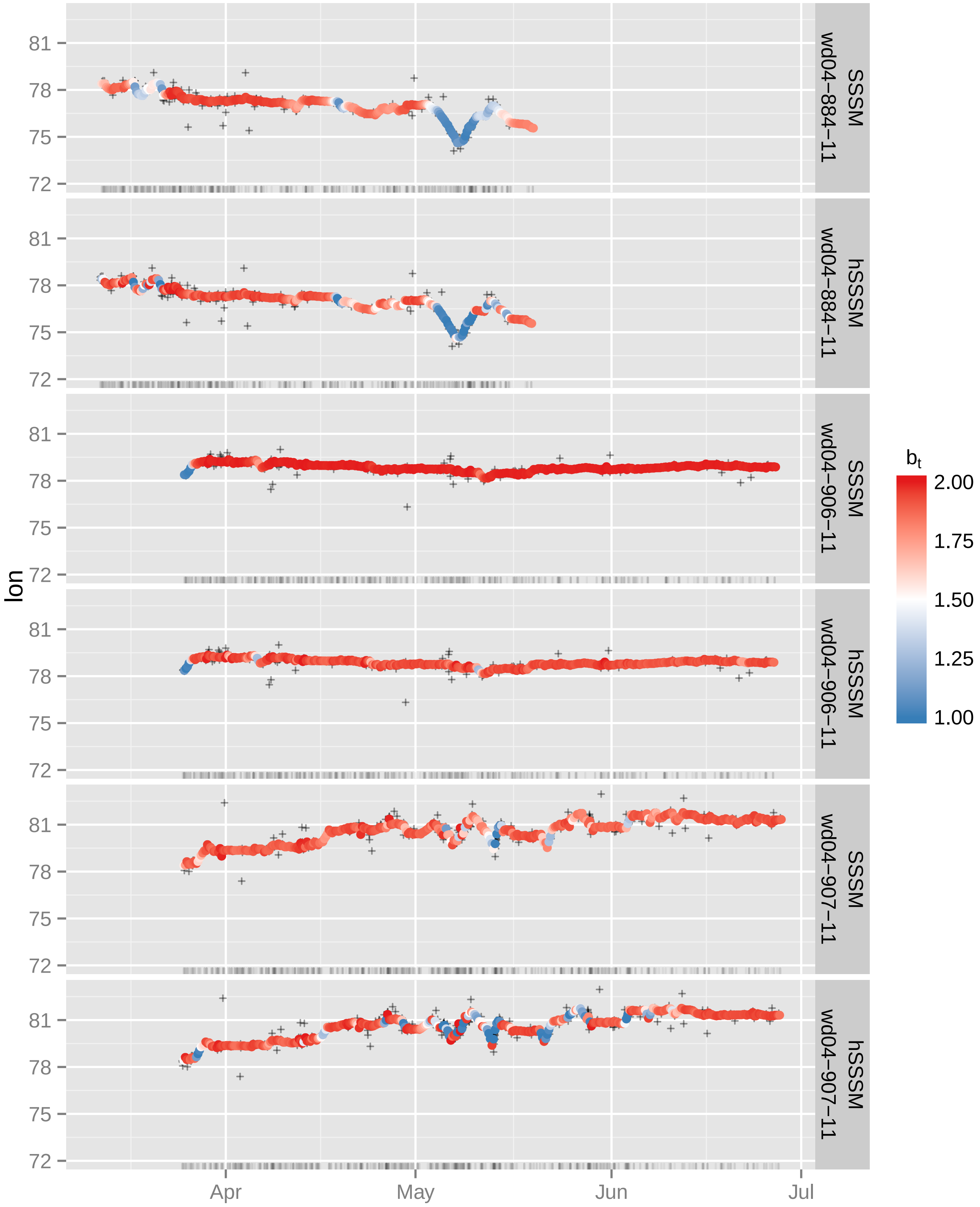

Figure S3 cont'd: Posterior mean longitude time-series coloured by the posterior mean behavioural state for the 8 seals not included in Fig. 3

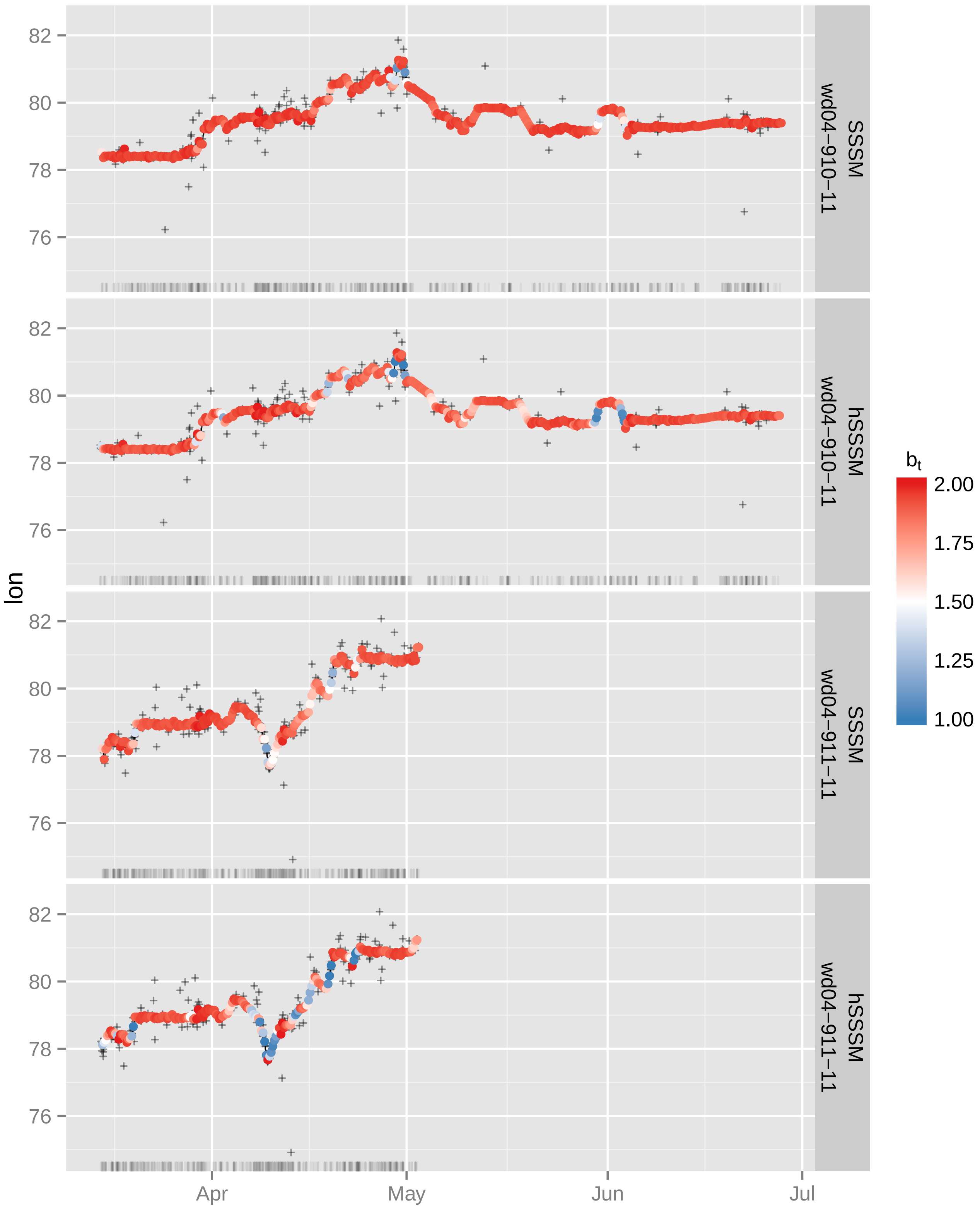

Figure S3 cont'd: Posterior mean longitude time-series coloured by the posterior mean behavioural state for the 8 seals not included in Fig. 3
